# The Story of the Insane from Year to Year

**Published:** 1889-06-01

**Authors:** 


### The Story of the Insane from Year to Year.

Asylum Reports.

### BRISTOL ASYLUM FOR THE CITY AND COUNTY OF BRISTOL.

This asylum has been added to and altered during the year to a very considerable extent. Dormitories for 428 patients have been built, and dayrooms for 84. Old dormitories have been converted into dayrooms, and still further additions and alterations are shortly to be made to the administrative block. One hundred and sixty-nine patients were admitted, 65 were discharged, and 60 died. In 36 of the admissions, the disease was hereditary, and in 6 only drink was the assigned cause. At the close of the year there were 484 patients in residence. It is worthy of note that 117 were epileptics. The recoveries were 36·9 per cent. on the admissions, and the death-rate was 13·2 on the average number resident. Boroughs have only one asylum rate, under which everything is charged. Counties have two rates—"maintenance" and "building." The average weekly cost at Bristol was 8s. 10½d., calculated in the way usual in counties. Dr. Thompson says: "After seventeen years of experience in this asylum, I have come to the conclusion that consumption is the most curable disease which we have to treat. In many cases privation previous to admission is doubtless the cause of this special disease, as it is of the mental state; and good feeding, no doubt, and a change of circumstances all round for the better has much to do with our success in arresting the disease, or in curing it altogether." It is a pity Dr. Thompson is not in private practice, or undoubtedly his consulting-room would be besieged from morning to night. Nevertheless, we quite see the point, and we are aware that consumption is a disease often arrested in our county asylums. The Anti-Tobacco Society would do well to quote the paragraph on page 12. We agree with Dr. Thompson, and regret want of space prevents us from quoting what he says. There are two assistant medical officers. It is pleasing to note that the stoker retired on a pension of two-thirds of his wages.

### EAST RIDING ASYLUM, BEVERLEY.

A rather prettily situated asylum, looking across Beverley Common and down on the old minster. On the last day of the year there were on the books the names of 291 patients, a goodly sprinkling of whom were private patients. During the year 69 new cases were admitted, 24 were discharged, and 22 died. The recoveries are rather low, standing at 28·12 on the admissions, while the average since the opening of the asylum is 29·45. The death-rate is low also, being 7·82 against an average of 10·71. Hereditary tendency caused the insanity in 13 instances, and drink comes close to it with 12. Cerebral disease was the cause of death in 13 cases, and phthisis figures as the cause in 3. The weekly cost per head was 8s. 6d., but the unions were charged 8s. 9d. Out-counties are charged 15s., and private patients pay from 13s. to 30s. The East Riding patients increased during the year by 26, an alarming increase for so small an asylum, but, as Dr. Macleod points out, this increase is not wholly owing to an increase of admissions, but partially to a low death-rate.

### GOVAN PAROCHIAL ASYLUM.

England has no exact counterpart to these Scotch Parochial Asylums. They would seem to be arranged for the reception of patients from certain parishes only. Govan is now a suburb of Glasgow, and the asylum is situated at Merryflatts. It contained at the end of the year only 240 patients. There were 98 admissions, 69 discharges, and 27 deaths. The recoveries were 41·8 per cent. of the admissions, and the deaths were 11·2 on the average number resident. Hereditary tendency, drink, and religion account respectively for 12, 11, and 4 of the admissions. Bronchitis was the cause of death in 4 cases, and phthisis in 2. Dr. Watson has no assistant medical officer. We did not notice any statement as to the weekly rate.

### GLASGOW ROYAL ASYLUM.

When the year 1888 went over to the majority, this institution had 480 patients in residence, being a decrease of 10 in the numbers for the previous year. Applications for the admission of paying cases were unusually numerous, and the contract for pauper cases had to be reduced from 200 to 175. From the North and the South, and the East and the West, we have the same cry about the unfavourable type of the cases admitted. Dr. Yellowlees joins in the chorus, and certainly with some reason, for we learn that 22 of the admissions were over 65 years of age, and 32 had been insane for upwards of a year before admission. When will the profession and the public become convinced of the enormous advantage of early treatment in mental diseases? Notwithstanding the above facts, the recoveries stand at 37·1 per cent., and the death-rate is only 5·4. Dr. Yellowlees's report is a very short one; but it is supplemented by a synopsis of the early history of the asylum, read at the meeting of the British Medical Association in Glasgow. Everyone acquainted with the institution will cordially endorse the concluding sentences of the paper: "The benevolent exertions of the founders of the Glasgow Royal Asylum have thus borne noble fruit. The institution has been an unspeakable blessing to multitudes, and age has not lessened its efficiency and usefulness."

### SOMERSET AND BATH LUNATIC ASYLUM.

With the year 1888 this asylum completed the forty-first year of its existence, and it had increased its numbers from 233 in 1848 to 804 in 1888. During the last year 216 patients were admitted, 69 were discharged recovered, and 100 died. The percentage of recoveries would stand at 33·6, and the deaths at 13·5 among the men and 11·4 among the women. A good many minor improvements seem to have been carried out during the year, and we should infer from the general tenour of the reports that Dr. Wade is a most energetic superintendent. On the vexed problem of asylum attendants and nurses he has the following well put remarks: "I am becoming more firmly convinced every day that the really best way to secure good attendants and to retain them, is, while duly paying every reasonable attention to their comfort, and making their situations worth keeping, to refuse all application for attendants' places from persons who have served in other asylums. The 'rovers' seldom stay anywhere long, and they unsettle many who would not otherwise change. Good attendants seldom change. Those who leave for 'a change' are not often worth retaining, and are no acquisition to any other asylum." There certainly cannot be a doubt of the truth of the above. The principle is acted on in many of our best English asylums, and we hope Dr. Wade's words will find an echo in the hearts of the non-contents.

---

Religious Consolation.—Do not overlook the desire for spiritual advice and consolation which patients sometimes feel, and, with the frightful *mauvaise honte* peculiar to Protestantism, alone among all human beliefs, are ashamed to tell. Especially in the higher walks of society, where this unutterably miserable false shame of Protestantism acts in proportion to the general acuteness of the cultivated sensibilities, let no unwillingness to suggest the sick person's real need suffer him to languish between his want and his morbid sensitiveness. What an infinite advantage the Mussulmans and the Catholics have over many of our more exclusively spiritual sects in the way they keep their religion always by them and never blush for it! And besides this spiritual longing, we should never forget that "on some fond breast the parting soul relies," and the minister of religion, in addition to the sympathetic nature which we have a right to demand in him, has trained himself to the art of entering into the feelings of others.—*From* Elsie Venner, *by Oliver Wendell Holmes.*

